# Communication and cooperation challenges in the online classroom in the COVID-19 era: a qualitative study

**DOI:** 10.1186/s12909-023-04189-1

**Published:** 2023-03-30

**Authors:** Shahin Salarvand, Masoumeh-Sadat Mousavi, Majid Rahimi

**Affiliations:** 1 Hepatitis Research Center, Faculty of Nursing and Midwifery, Lorestan University of Medical Sciences, Khorramabad, Iran; 2 Nursing and Midwifery Care Research Center, Isfahan University of Medical Sciences, Isfahan, Iran; 3 Department of Health Education and Promotion, Health Faculty, Isfahan University of Medical Sciences, First Floor, No. 47, Hajian Lane, Simin St., Isfahan, 81769-35747 Iran

**Keywords:** Communication, Online Classroom, COVID-19 era

## Abstract

**Background:**

The increasing prevalence of COVID-19 affected all aspects of life, including education. Communication and interaction are vital in any form of education. This study explained health profession educators’ and students’ experiences regarding the challenges of communication and cooperation in exclusively online classrooms during the COVID-19 era.

**Methods:**

The present descriptive explanatory qualitative study examined health profession educators’ and students’ experiences with exclusively online classrooms during the COVID-19 era. They were included in the study by purposive sampling. In-depth and semi-structured telephone interviews were conducted to collect data. The content analysis presented by Graneheim and Lundman was used to analyze the data. The present study employed four strength criteria: credibility, confirmability, transferability, and dependability.

**Results:**

The results of the present study included communication and cooperation challenges in exclusively online classrooms related to the COVID-19 pandemic. Two themes emerged from 400 open codes: lack of students’ socialization and communication-related concerns, which each had subcategories.

**Conclusions:**

Lack of students’ socialization and communication problems were identified as the participants’ main experiences. Defects in teacher training due to the sudden transition to virtual education, acquiring a professional identity that is possible in in-person education was also flawed. The participants experienced challenges in their class activities, leading to a decrease in trust, a lack of motivation to learn from students, and teachers’ teaching. Policymakers and authorities should adopt new tools and techniques to improve exclusively virtual education outcomes.

## Background

The increasing prevalence of COVID-19 affected all aspects of life, including education [1]. The closure/lockdown policy to reduce individuals’ interactions was a way to cope with such a situation [2]. Numerous medical schools suspended clinical classes and activities in the hope of reducing the disease incidence [3]. This lockdown emerged as the major educational challenge in the last 50 years [4]. Most governments employed alternative methods to educate students [2] in order to maintain the continuity of education [5]. Thus, virtual education was a method that provided educational content to learners in a certain time and place [6].

Although changes in the educational approach to virtual education had been already begun [7], this method was used more rapidly due to the onset of the pandemic [5]. The prevalence of COVID-19 and then the closure of universities and the sudden shift to virtual education worldwide revealed numerous conceptual, educational, and technical gaps. Each university as an educational system reacted within the framework of its limitations [8]. Owing to the closure of educational centers in Iran, the educational system authorities also found themselves obliged to use various software programs and tools to follow the education flow via the cyberspace; however, they encountered serious challenges due to the lack of necessary infrastructures and facilities in terms of design and implementation [9], and the way of virtual teaching [10].

This type of education is not well-established in Iran, and its achievements are not yet tangible [11]. A literature review indicated a number of concerns about the sudden use of virtual education, such as the impact on the academic process, lack of interaction between teachers and learners, large volume of educational content, reduced motivation for teaching and learning between teachers and learners, lack of skills in the use of technology, lack of necessary equipment, problems and costs of the Internet, place of study, participation rate, compatibility rate, satisfaction with the format of online classes, and study time in the online education process [12, 13]. In addition, professional socialization is a major concern, especially in medical students, which has been disrupted during the COVID-19 era [14].

Virtual education also has benefits such as the freedom to choose time and place, access to 24-hour educational content, no need to commute especially during pandemics and reduced related costs, learners’ independence and responsibility for learning, a safe learning environment, increased awareness about personal capabilities, and work with computers and related programs [7, 12]. In another study in Iran, students were dissatisfied with virtual education for various reasons such as lack of infrastructure, non-standard content, communication problems, and lack of feedback [15].

The perception of the stakeholders’ points of view, including students and health profession educators is crucial to enforce education policies [20], and obtain a holistic view of the phenomenon. Numerous qualitative types of research have reported different stakeholders’ understanding of the same phenomenon [21–24] Furthermore, they have relatively the same experiences. In addition, it is necessary to obtain varying perceptions of the cooperative roles that different stakeholders should take in an exclusively online classroom [21].

Online learners need to be motivated to overcome the challenges of procrastination due to online learning [25], and use online communication facilities to create meaningful interactions [26]. Therefore, there is a positive and significant relationship between interaction dynamics, learning outcomes, and student satisfaction, indicating the importance of teacher-learner and learner-learner interactions [18]. Although most published literature discusses the benefits of virtual education, their challenges during virtual education should be considered [27]. Since education is a student-teacher interaction, and traditional education immediately changed to virtual education in Iran, the stakeholders’ experiences in the process of quality teaching and learning are important. The present qualitative study explained health profession educators and medical sciences students’ experiences regarding the challenges of communication and cooperation in online classrooms in the COVID-19 era.

## Methods

The present descriptive exploratory qualitative study examined health profession educators and students’ experiences with communication in exclusively online classrooms during the COVID-19 era, and they were included in the study by purposive sampling. The purpose of a qualitative study is not to randomly select people to manipulate, control and generalize the findings, but to understand more regarding the phenomenon under study [28]. Numerous qualitative types of research report different stakeholders’ understanding of the same phenomenon [29].

Education (including virtual education) is a phenomenon formed based on the interaction [30], and communication [31], between the learner and the instructor. In this study, virtual education during the COVID-19 era, which was an emerging phenomenon in education in Iran, was investigated. Owing to different perceptions of the common roles of stakeholders in education [21]; therefore, people involved in virtual education, including health professions educators and students, were selected to participate in the study for these reasons; (1) It was expected that the participants’ experiences would be the same. (2) It was necessary to acquire a comprehensive image of the phenomenon, since the maximum variation sampling can be used to build a comprehensive understanding of the phenomenon [32]. In addition, one kind of triangulation refers to the use of multiple data sources or data collection from different persons (i.e. individuals, groups, etc.) in qualitative research to make an overall understanding of a phenomenon [33, 34].

The participants were included in this study by purposive sampling. Sampling was performed on participants with the maximum variation in age, sex, field of study, and year of education/work experience. The inclusion criteria for the participants were to have experience with virtual classes for at least one academic semester, and willingness to participate in the study.

Deep and semi-structured telephone interviews, due to the conditions of the COVID-19 pandemic, from October 2021 to January 2022, were used to collect data. According to the research objectives, the participants’ characteristics were as follows: students and health profession educators of the University of Medical Sciences with the consent to participate in the research and to express their experiences. The participants were selected from different faculties of Isfahan University of Medical Sciences, including the faculties of medicine, nursing, and midwifery, dentistry, pharmacy, health, nutrition, and rehabilitation. The participants’ age range was 21 to 29 years for students and 34 to 51 years for health professions educators. The level of study was undergraduate, master’s, and doctoral for students. The academic level of the health profession educators was assistant professor, associated professor, and professor. The students and health profession educators’ education and work experience were 3 to 7 and 4 to 27 years, respectively.

Before the interviews, the conditions of conducting the interview, including explanations of the objectives, optional participation in the interview, confidentiality of the participant’s profile, recording of the participant’s voice, and commitment to provide the research results upon their request, were explained. Then, the participants signed written informed consent forms electronically and we asked them to determine the time of the interview to be in a calm and convenient environment. We interviewed them after they agreed.

The main question asked from both groups in the study was “What is your experience of communicating and cooperating in online classrooms during the COVID-19 era? The next questions continued according to the participants’ statements to obtain deeper information (Table 1).

**Table 1:** **The topic guide of study/questions asked from health profession educators and students during the interviews**

Questions	Participants
What is your experience of communication in exclusively virtual classrooms during the COVID-19 lockdown?	Health profession educators
What is your idea about interaction with students in virtual classrooms of the COVID-19-induced lockdown?	
How the communication has been affected in virtual classrooms of the COVID-19-induced lockdown?	
Please explain more….	
What is your experience of communication/cooperation in exclusively virtual classrooms during the COVID-19 lockdown?	Students
What is your idea about interaction with health profession educators/other students in virtual classrooms of the COVID-19-induced lockdown?	
How the communication/cooperation has been affected in virtual classrooms of the COVID-19-induced lockdown?	
Please explain more……	

## Data collection

The interviews lasted about 20 to 60 min. The third author (M.R.) conducted the interviews and recorded all interviews with a voice recorder according to the participants’ consent. The second author (M.M.) transcribed the recorded interviews verbatim. The first author analyzed the interviews, and the second author revised the codes and the coding process. The next interview was conducted after analyzing the previous interviews and extracting the exact codes. When conducting the interviews, the interviews were also analyzed to achieve data saturation with 21 samples. We also asked an expert in qualitative research (N.B.) to review the coding and analysis process.

Data were analyzed simultaneously with the collection. The content analysis presented by Graneheim and Lundman was used to analyze the data [35]. At the end of each interview, the participants’ recorded voices were repeatedly listened to, their statements were transcribed verbatim, and each transcribed interview was read several times to understand their experiences and perceptions. The data with relevant meanings and statements were underlined, and thus meaningful units were identified. Each meaningful unit was summarized into a condensed meaningful unit, and the initial codes appeared. A total of 400 initial codes were extracted from the interviews. S.S. carefully studied the initial codes and classified them as subcategories according to the similarity of the concept. During this inductive process, similar subcategories were classified as categories, and then similar categories developed themes. This process of coding and the emergence of the themes were reviewed and discussed with S.S. by the second and third researchers. Finally, the themes were determined as an expression of the hidden content of the text. Interviewing was discontinued by data saturation. The data saturation was separately achieved in each participant group. (10 health profession educators and 11 students).

### Rigor

The present study employed four rigor criteria, including credibility, confirmability, transferability, and dependability, as recommended by Lincoln and Guba [36]. The credibility of the findings was enhanced by investigating the transcripts, examining the agreement between the two coding processes by two independent persons, and validating the findings with the participants [37]. For credibility, the extracted codes were referred to the participants, and the findings were validated by their approval (member check). The researcher referred the extracted findings and codes to an expert in qualitative research, and the expert (peer check) confirmed the validity of the research findings. The confirmability was achieved by bracketing (excluding preconceptions), reporting, and recording the research steps and decisions accurately, so that others could follow it if desired and the audit trail could be performed on research.

For the dependability of the findings, more than one researcher conducted the data analysis process. Transferability was provided by various samples (health profession educators and students) from different faculties and demographic characteristics.

## Results

The results of the present study included communication and cooperation challenges in the online classrooms related to the COVID-19 pandemic in two themes as follows: lack of students’ socialization and communication-related concerns, which each had subcategories (Table 2).

### Lack of students’ socialization

In the present study, the participants noted the lack of medical students’ socialization in mere virtual education during the COVID-19 pandemic. This theme contained five categories, namely decreased sense of social presence and classroom management, decreased desirable social learning, lack of professional identity acquisition, inadequate educational feedback, and infrastructural barriers to establishing desirable communication.

Decreased sense of social presence and classroom management had subcategories, namely reduced student participation in the classroom, and poor management of students. The participants also mentioned the reduction in student participation in the classroom that led to a decrease in a sense of community and poor management in the classroom.

*“Students do not participate well, and the lack of proper feedback from students disrupts class communication.” (T.10)*.

The participants stated that it was impossible to manage students well due to the lack of observation, which it could lead to decrease the student participation in the classroom.

*“It is very difficult to manage students in virtual classrooms, because we do not know whether they are present or they are doing other activities at that time when we see their names on the page.” (T.9)*.

They also noted a decrease in desirable social learning, which had subcategories, such as a diminished role of the health profession educator in teaching, a decrease in cooperative learning, and a decrease in students’ motivation to learn. The participants expressed a diminished role of the health profession educator in teaching:

*“Despite health profession educators’’ efforts in teaching, it seems that their roles in teaching have diminished and students should make more efforts to understand the lesson content.“(St.6)*.

They also stated a decrease in cooperative learning and cohesiveness due to the lack of effective communication in the online classroom:

*“The worse thing was that in this period, we had no enthusiasm, for example, before the pandemic period, and it was suitable in the university environment with other students, because we were interacting and talking to each other about a subject. The content remained in our minds for a longer period and thus face-to-face education was much better than the virtual one (St. 8).“*

The participants reported a decrease in students’ motivation to learn and sense of belongingness that could led to students’ academic failure with numerous consequences.

*“Some of the students with whom I had a face-to-face classroom, unfortunately, have an educational decline now. This educational decline was obvious. The students complained of poor learning. The students, for whom studying was important and those, who were not very sensitive to studying, also said that they could not establish a proper communication with health profession educators.” (T.2)*.

The participants reported a lack of acquiring professional identity containing subcategories, including lack of health profession educators’ role modeling and lack of the hidden curriculum.

*“The university has a duty to humanize, and the presence of students in the university and its atmosphere can affect their behavior in actions and reactions in society. Their absence in the university can also be a weakness for health profession educators and students” (St.3).*

*“As long as students do not see health profession educators, they will unfamiliar with a professor’s character. In my opinion, it is very effective in modeling for students, especially freshmen during the COVID-19 era, who have no perception of the faculty and student or health professions educator behavior, and the role modeling of a professional person.” (T. 4)*.

*“This reduction in interactions and face-to-face/in-person communication led to disappearance of hidden learning and curriculum, and diminished the roles of social learning and role modeling.” (T.7)*.

They also reported a failure of educational feedback as a subcategory of students’ lack of socialization, which included the following subcategories: one-way communication or lack of two-way communication, lack of interaction with students, offline feedback on student assignments, student-health profession educator interaction via different platforms, and unfair evaluation.

*“In virtual education, communication is often one-way, and the possibility of getting feedback decreases due to the high number of students.” (T.9)*.

*“Compared to in-person classrooms, where we can see all students, we do not know if students who are seemingly online, have a really active presence or not!“ (T.10)*.

*“We give feedback on students’ assignments on the presenting website of lesson content (NAVID; a learning management software program) and review the quality of the assignments by them.” (T.9)*.

*“There are several platforms through which we hold classrooms.” (T.2)*.

As mentioned above, the participants expressed unfair evaluation:

*“I think the evaluations are weak, and we cannot implement all the components of a proper evaluation.” (T.7)*.

They also pointed out infrastructural barriers to establishing optimal communication, including subcategories such as student’s economic status, slow Internet speed, lack of appropriate facilities, and lack of a support center.

*“Sometimes, we see students living in remote villages or are economically poor and unable to communicate well in virtual classrooms due to very low Internet speed or lack of smartphones or equipment.” (T.6)*.

*“…The slow speed/frequent disconnection of the Internet could be a barrier in virtual communication; therefore, we cannot timely/properly listen to or see each other.” (St.8)*.

*“We are not well equipped for launching an online classroom.” (T.9), (St.2)*

*“Another problem is the lack of a support center for the system. Sometimes, the system has a problem and, as a health profession educator, I cannot enter the classroom or there is a voice problem, and there is no one to fix the problem.” (T.2)*.

### Communication-related concerns

This subcategory contains some subcategories, including uncivilized behaviors, feelings of lack of support and encouragement in students, and dilemmas.

In the subcategory of uncivilized behaviors, the participants reported the unfamiliarity of the parties with each other and making misunderstandings, academic dishonesty, concealment, procrastination, and lack of privacy/confidentiality during the COVID-19 pandemic virtualization course.

*“One of the weaknesses of virtual education, in my opinion, maybe because some learners do not know each other and do not see each other, then they may be embarrassed to ask health profession educators some questions if they did not understand something. On the contrary, in in-person education, health profession educators can get feedback, for example, to what extent the content which they are teaching now, both theoretically and practically, is understood, to what extent they should add and say less, for example, add in a certain subject, but not in virtual education, because they are far from each other and do not see each other, and the body language areas are ignored. In face-to-face education, especially practical lessons, if students do not understand something, they can ask questions to resolve the ambiguity or see and understand better rather than watching movies online (T. 7).*

*“You know the problem is that once the connection is lost, then the health profession educator thinks you did not listen, for example, if you ask a question, you say, for example, I did not understand this part, it was disconnected, then the health profession educator thinks that you did not listen from the beginning. For instance, the health profession educator gives you a negative point. It has happened and been difficult to prove.“ (St. 2)*.

Participant 3 reported the impossibility of managing students and their approach to doing assignments that increased cheating in the presentation of assignments.

*“Most students cheat on homework or exams online/offline and get fake grades”. (T.3)*

*In the activities, which we did or heard from other people, and the assignments we sent, we might get the same answers, indicating cheating in the presentation of that assignment; however, if it was in in-person classrooms, question asking and students’ answers could help them to learn better than just copying and pasting assignments.” (St.9)*.

*“In online classrooms, some students either did not activate the camera or were not present, and her/his name was online, and when we called their names, they stated that the microphone had a problem, and they could not announce their presence”. (T. 6)*

*“In offline classrooms, there was no need to be active and they even copied the assignments or delayed doing the assignments or giving feedback”. (T.1)*

*“An issue in cyberspace and virtual education is the lack of privacy and confidentiality of people like sharing the health profession educator’s photos or the content presented in the classroom in cyberspace.” (St.6), (T.4)*

In the subcategory of the feeling of lack of support and encouragement in students, the participants of the present study pointed out the double effort of students, the forced student-centered approach, and lack of motivation due to lack of communication and interaction with health profession educators and other students.

*“The absence of students in the classroom atmosphere causes them not to study very well as the university is not just an environment for them to study. We go to university to see friends and talk to them, and everything is now diminished, and just study is left, so we should try harder to learn the lessons. Well, it gets a little hard and its sweetness no longer exists”. (T.7)*

*“When the lack of interaction increases over time, the individuals’ spirit is considerably affected, that is, they may not understand it, but when their social relations decrease, it is very effective in their spirit.“ (St. 10)*.

In the sub-category of dilemmas, the participants expressed the reduction of educational innovation, health profession educators’ distress, students and health professions educators’ procrastination, and the high volume of dialogues.

*“In my opinion, it was one of the worst disadvantages. In practice, this interaction became one-sided, and a classroom, which could now become dynamic, became summarized. Furthermore, several classrooms were offline, and health profession educators recorded their speeches. The offline classrooms made health profession educators tired, so they left them, and it might happen even for students”. (T. 5)*

In the present study, health profession educators experienced distress and extra workload. Participant (T.10) stated that communication in an online classroom was difficult as if “a wall in front of you blocked your attempts at communication.”

*“In my first virtual classroom, I had approximately 50 students, and it was a specialized classroom. It was one-sided and I could not see the faces of the students and had no feedback, so in the middle of teaching, I forgot what I was teaching, I was very, very tired than the time in face-to-face classrooms as if it was a wall in front of you and it was very painful.“ (T. 10)*.

*“…I hold all of my classes online, not face-to-face, and thus I did not even leave the voice for any classrooms as offline sessions, and they were exactly like the atmosphere of my face-to-face classrooms as I asked questions and waited for their answers, and then gave assignments to them. If they had questions, I tried to explain.” (T.8)*.

The participants also stated the procrastination of some students and health profession educators in establishing desirable communication under such conditions.

*“Some students and colleagues procrastinated in various ways and did not communicate well to enhance learning.” (T.2)*.

Health profession educators stated that they encountered many dialogues/chats in online teaching, as answering them disrupted the teaching process, and it was difficult for the health profession educator.

“*There were a large number of students, and thus, there were many dialogues during the online classroom, which we could not respond them effectively. Even recently, the even-odd plan was created to halve the number of students with the hope of increasing the health profession educator-student interaction.” (T. 1)*.

**Table 2:** Subcategories, categories, and themes of data analysis

Subcategories	Categories	Themes
Decreased student participation in the classroom	Decreased sense of social presence and classroom management	Lack of medical students’ socialization
Poor management of students		
Diminished role of the health profession educator in teaching	Decreased desirable social learning	
A decrease in cooperative learning and cohesiveness		
A decrease in students’ motivation to learn and sense of belongingness		
Lack of health profession educators’ role modeling	Lack of professional identity acquisition	
Lack of the hidden curriculum		
One-way communication/ Lack of two-way communication	Inadequate educational feedback	
Lack of interaction with students		
Offline feedback on students’ assignments		
Student-health profession educator interaction via different platforms		
Unfair evaluation		
Students’ weak economic status	Infrastructural barriers to establishing optimal communication	
Slow internet speed		
Lack of appropriate facilities		
Lack of support center		
The parties’ unfamiliarity with each other and creating misunderstandings	Uncivilized behaviors	Communication-related concerns
Academic dishonesty		
Concealment		
Procrastination		
Lack of privacy/confidentiality		
The double effort of students/ the forced student-centered approach	Feeling of lack of support and encouragement in students	
Lack of motivation due to lack of educational interaction with other health profession educators and students		
The reduction of educational innovation	Dilemmas	
Health profession educators’ distress		
Students and health professions educators’ procrastination		
The high volume of dialogues		

## Discussion

The present study aimed to explain the experiences of health profession educators and students regarding the challenges of communication and cooperation in the classroom during the COVID-19 era. Overall, the results showed that the problems caused by virtual education outweighed its benefits. Issues like lack of participation and communication of students in the class, problems of class management, incomplete infrastructure and insufficient training of professors due to the speed of transition to virtual education, lack of motivation of professors and students, lack of innovation, and incorrect assessment of students were among the biggest problems. The research results included two themes of health profession educator-student communication experiences in the COVID-19 era as follows: Lack of students’ socialization, and communication-related concerns, which each theme had subcategories.

### Lack of students’ socialization

The first theme is the lack of students’ socialization consisting of five subcategories as follows:

Decreased sense of social presence and classroom management, decreased desirable social learning, lack of acquiring professional identity, inadequate educational feedback, and infrastructural barriers to establishing optimal communication.

#### Decreased sense of social presence and classroom management

The participants in the present study experienced a decreased sense of social presence and classroom management, which included lower student participation, and poor student management.Other studies confirm the findings. The sense of the presence of teachers and learners is a challenge in establishing the student-teacher interaction in the virtual classroom [40]. The results of one study by Kunaviktikul indicated that students felt social isolation due to forced social distance and lockdown laws that led to the closure of schools [14]. It caused their socialization was impaired [39]. So, cognitive social presence and affective social presence were not fully established [38]. A positive learner-teacher interaction in increasing the learner participation in the virtual classroom can be challenging due to the lack of social and emotional support [42]. We think the decrease in students’ motivation and sense of belonging can effect by the lack of social presence and being unknown to others which have an interchangeable relation to lack of social activity. For better classroom control, management should encourage their health profession educators to increase their skills and knowledge in online education [38] [44].

#### Decreased desirable social learning

The present study showed that the participants expressed a decrease in optimal social learning. The results indicated the diminished roles of health profession educators in teaching. Xu et al. reported that the online chat space limited the potential of various learning activities and educational strategies, and there was no possibility of a close emotional relationship with the teacher’s facilitation strategies [45].

The results of the present study indicated a reduction in cooperative learning in the classroom. Sawal et al. reported a very weak general relationship between knowledge-sharing behaviors and online socialization in the e-learning environment [46].

In the present study, the participants reported a decrease in students’ motivation and sense of belongingness to the classroom. Sweeney et al. reported that anonymity in online communication may reduce students’ sense of belonging [48].

#### Lack of acquiring professional identity

The participants of the present study experienced lack of professional identity acquisition. On the contrary, one study by Kunaviktikul indicated that online education caused the professional encouragement and personal development of some participants via digital software [14]. The participants of the present study pointed out the weakened role modeling and shortcomings in the hidden curriculum in this subcategory. Dedeilia et al. in their study showed that students would learn some content through the hidden curriculum, see the teacher, and learn a professional role. Eye contact and body language are basic educational principles that are generally or partially inaccessible in virtual education with current facilities [49]. Therefore, the exclusively online educational communication causes medical students to be deprived of the benefits of health profession educators’ role modeling, social learning, and hidden curriculum effects.

#### Inadequate educational feedback

In the present study, the participants reported failure in educational feedback, including one-way communication/lack of two-way communication, lack of interaction with students, feedback on student assignments, student-health profession educator interaction via different platforms, and unfair evaluation. Numerous studies confirmed these findings and indicated no or insufficient interaction and educational feedback in online classrooms [14, 50, 51], and feedback and evaluations were unfair [14].

#### Infrastructural barriers to establishing optimal communication

In the present study, the participants pointed out infrastructural barriers to establishment of optimal communication in the exclusively online classroom, including student’s economic status, slow internet speed, lack of appropriate facilities, and lack of a support center. Keshavarzi et al. confirmed that the virtual education infrastructure was improper, and some disruptive factors hindered its implementation, including slow Internet connection, lack of equipment, necessary time, and high cost of virtual education [52]. Other studies demonstrated that the participants living in rural areas and/or at a lower socioeconomic level experienced more learning difficulties [14, 53]. Several other studies also reported weaknesses in infrastructures such as slow Internet speed and internet connection problems as barriers to the use of virtual education [27, 54].

### Communication-related concerns

The second theme is communication-related concerns consisting of three subcategories as follows: uncivilized behaviors, feelings of lack of support and encouragement among students, and dilemmas.

#### Uncivilized behaviors

The participants reported uncivilized behavior, including the unfamiliarity of the parties with each other and creating misunderstandings, academic dishonesty, concealment, procrastination, and lack of confidentiality and privacy.

This study reported the participants experienced unfamiliarity with each other and making misunderstanding between the parties. Ahlers, et al. reported that students mentioned not knowing other participants as one of the reasons for not participating in classes [55]. It appears the exclusively virtual communication may lead to misunderstandings between the health profession educators and learners.

The present study indicated the high frequent academic dishonesty among students. The findings of other studies confirmed an increase in the prevalence of cheating behaviors and academic dishonesty in the form of illegal collaboration in online classrooms among students [56, 57]. Furthermore, Keshavarzi et al. reported that unauthorized copying of uploaded contents and copyright infringement occurred in the virtual education environment, being a clear example of legal challenges and academic dishonesty in the field of virtual education [52].

In the present study, the participants reported concealment by students. Other studies also confirmed and indicated that it was difficult to detect students’ absenteeism in a virtual classroom, since they might be present and become online, and their names might be shown on the attendance list; however, they might surf the web or engage in other activities [58, 59]. In other words, they might be absent physically and mentally.

The present study indicated students and health professions educators’ procrastination., Melgaard et al., reported procrastination of class by health profession educators and students was another concern of virtual education [60]. Procrastination was experienced in online and offline teaching, and it was another motivational challenge in online learning [58]. Song et al. also found that procrastination was easier in online learning situations than in traditional face-to-face classrooms [61]. It appears there are several predictors for procrastination, as Jia et al., study showed during the COVID-19 pandemic, procrastination caused anxiety among medical students. This was due to isolation, lack of attendance in class and lack of proper feedback from teachers [62]. The other participants’ experience was that the students did not observe confidentiality and privacy; therefore, some students shared the teacher’s photos or the content presented in the classroom in cyberspace. The study by Rajab and Soheib reported that most respondents preferred not to use webcams during online academic activities due to confidentiality, anxiety, stress, and fatigue [63]. Additionally, the results of the study by Lassoued et al. showed that data security and confidentiality were identified as a concern [64]. This issue can cause a challenge in online educational communications.

#### The feeling of lack of support and encouragement in students

In the present study, the participants experienced a sense of lack of support and encouragement in students, which included two subcategories: double student effort/the forced student-centered approach and lack of motivation due to lack of communication/interaction with health profession educators and other students. The students also made more efforts to better understand the content to compensate for the shortcomings of virtual communication in teaching. Other studies confirmed that virtual education was suitable for self-centered learning [51, 61]. Srivastava et al. also reported that students experienced lower motivation to study and increased pressure for self-directed learning [65]. In fact, they tried to develop and keep motivated. As self-directed learning increases students’ motivation and autonomy [66]. Although this issue challenged the students, it pushed them to the forced student-centered, which in some students can make personal development.

The present study indicated that the students reported a lack of motivation due to a lack of educational interaction with health profession educators and other classmates. The results of one study by Kunaviktikul et al. indicated that students felt higher levels of mental distress and loneliness and were less motivated and concentrated on online learning [14]. Without a face-to-face classroom, students felt as if they interacted with a computer instead of real people; hence, they found it difficult to be motivated in online courses where there was no social presence [67]. Arif et al. reported that the need to communicate with learners was neglected due to the limited time to provide teaching in virtual classrooms [40].

#### Dilemmas

This study indicated the participants experienced difficulties/dilemmas in exclusively virtual education during the COVID-19 pandemic. The decrease of educational innovations was one of these experiences. Armstrong et al. reported that there was an insufficient chance to solve queries and to clarify concepts for complex topics due to the lack of the student-teacher interaction in virtual education [68]. Therefore, educational innovation decreased owing to unresolved ambiguities.

The present study reported that health profession educators described the difficulty of online teaching. Other studies confirmed that teachers experienced difficulties in these conditions. The most common problem that the professors expressed in changing the teaching method was unfamiliarity with virtual education platforms [10]. Sher’s study stated online students were often geographically isolated from the academic community, thereby increasing teachers’ workload to facilitate online classroom interaction. If students are confused in an online space, there is no clue unless they decide to communicate about their confusion [18]. Kunaviktikul et al. reported that the lack of a two-way interaction decreased teachers’ motivation to continue their online teaching [14, 69]. Furthermore, Kim et al. found that students might not be interested in establishing communications with their teachers, thereby affecting teachers’ efforts to develop a meaningful relationship with learners. Creation of positive learner-teacher communication in increasing learners’ participation in the virtual classroom can be challenging due to the lack of social and emotional support [42]. The resources of educational programs should be used in a way that facilitates the possibility of changing the teaching method in virtual education [10].

## Conclusion

The transition from face-to-face to virtual classes occurred suddenly and unplanned during the COVID-19 pandemic, causing most of these communication challenges. Problems in students’ socialization and communication problems were identified as the participants’ main experiences. Lack of attendance in the classroom and face-to-face interactions led to the lack of effective feedback on learning. Acquiring a professional identity that is possible in in-person education was also flawed. In the meantime, the problem in the technological infrastructure caused the intensification of health profession educators’ worries and distress.

The communication barriers in this research were caused by the lack of sufficient knowledge of health profession educators and students about each other, which led to a decrease in trust, lack of motivation to learn in students, and health professions educators’ teaching. The prevalence of cheating among students caused the unfair evaluation of students and increased the mistrust in health profession educators and the lack of motivation in active students. In addition, the large amount of material presented in online classes was mentioned as another obstacle to students’ learning. Finally, the present study demonstrated the increase of informal student-student and student-health professions educator dialogues and virtual communication; however, the educational communication leading to improved learning was weak.

### Suggestions for policy-makers

This study stated that how the exclusively virtual education could affect on medical education. Although there was a special condition during the COVID-19 pandemic and we were forced to have exclusively online classrooms, we realized that it was better to have both virtual and in-person classrooms as blended teaching and learning under normal conditions.

Given to the existence of the limitations and challenges in exclusively virtual education, it is necessary to create a supportive environment and a sufficient infrastructure for students and health profession educators for exclusively virtual education, so that this process must continue with less stress and higher productivity as possible. The policymakers and authorities should create new tools and techniques to improve the exclusively virtual education outcomes. This special attention leads to more useful close interaction and recognition of health professions educators and students from each other. Also, it increases students’ motivation to learn, to be attractive classroom for students. It causes to reduce cheating and improves the fair evaluation process, asking and answering questions in cases of ambiguity by students and health professions educators, actively becoming students and getting feedback will be better. Determination of scientific resources appropriate to the courses offered prevents the creation of a large volume of materials and causes education to be more attractive for health profession educators and students.

## Data Availability

The datasets used and/or analyzed during this study are available from the corresponding author.
